# *Scandinavian Journal of Work, Environment and Health* goes full open access

**DOI:** 10.5271/sjweh.3916

**Published:** 2020-09-01

**Authors:** Reiner Rugulies, Alex Burdorf

**Affiliations:** National Research Centre for the Working Environment, Copenhagen, Denmark [e-mail: rer@nfa.dk]; Department of Public Health, Erasmus Medical Centre, Rotterdam, The Netherlands [e-mail: a.burdorf@erasmuscmc.nl]

As of 1 January 2021, the *Scandinavian Journal of Work, Environment and Health* (SJWEH) will become a full open access (OA) journal. Under the “gold” OA status, all new articles will be published as “unlocked content” on the journal’s website. This makes the final version of an article freely accessible for everyone, and allows for distribution and adaptation as long as the authors are duly acknowledged as they retain the copyright. Online subscriptions will no longer be needed and thus terminated, whereas subscriptions for the printed journal will continue.

Going full OA is a major step in the 45-year history of the journal. With this step, we join the rapidly growing Open Science movement (1). A core aim of this movement is “making full and immediate open access a reality” as proposed by “Plan S” (also known as “cOAlition S”), which has been endorsed among others by the European Commission, the World Health Organization and numerous funding agencies including the Academy of Finland, the Research Council of Norway, and the Swedish Research Council for Health, Working Life and Welfare (Forte) (2). OA publishing means that research results are immediately available both for critical discussion in the whole scientific community – including researchers in low income countries who may not have access to subscription journals – and for consideration and decision-making by stakeholders and policy-makers. During the current COVID-19 pandemic, many journals have pledged to publish COVID-19 related articles as OA, acknowledging the societal need of getting unrestricted, immediate access to research results (3). At SJWEH, we believe the societal need of getting immediate access to research results is not limited to times of a pandemic.

To become one of the first occupational health and safety journals to go full OA is a double-edged sword. On the one hand, we are proud to be a first mover and support the Open Science movement. On the other hand, there is an economic risk for the journal. Unlike other major journals in occupational health and safety, SJWEH is published not by a large commercial publishing house but by a not-for-profit organization: the Nordic Association of Occupational Safety and Health (NOROSH) (4). Thus, the journal is not published to make a financial profit, and we operate on a relatively small budget with the constant challenge to balance income and expenses. This does not leave much room for error and, therefore, we contemplated full OA for several years before taking the final step (5–7). After continuously increasing the journal’s OA content over the years, we now feel we are in a strong position to go 100% OA.

Under the gold OA model, authors are requested to pay an article processing charge (APC) of currently €2300 (NOROSH members: €1150) to publish their papers in the journal. The fee is waived for articles from research institutes in a low income country (according to the definition by the World Health Organization) (8), while articles from research institutes in a middle income country receive an 80% reduction. All articles are published under the Creative Commons CC-BY 4.0 license, the most liberal license that allows for the sharing and adaptation of material as long as proper credit is given to the original source (9).

OA increases the opportunity for article citations (10). The five most often cited articles in 2019 among those published in SJWEH in 2018 were all open access articles (11–15). Thus, OA also benefits authors.

Sometimes a concern is voiced that the gold OA and APC model may result in a decline of quality because journals might be tempted to publish as many articles as possible to generate income (16). Indeed, the ever growing list of predatory journals that send out mass emails to researchers pressing for submission of papers (constituting nowadays a substantial proportion of the daily emails researchers receive), and then publish the articles with no or only a superficial peer-review process, after cashing in on the APC, indicates that publishing poor research might be an attractive business model (17). In SJWEH, though, this will not happen. Our Associate Editors are among the finest scholars in the field of occupational health and safety and they will continue to ensure that only high quality papers are sent out for peer-review, which is – in turn – ­performed by leading experts in the field. In other words, we can promise our NOROSH members, authors and readers that it will remain difficult to publish papers in the journal and that only the best papers will make it.

The Open Science movement is an exciting development transforming the way research is communicated and shared. We are proud to be a part of this movement, and we ask our authors, reviewers and readers to support the advancing of scientific knowledge in the field by continuing to send high quality papers to the journal, performing rigorous peer-reviews, and reading and discussing our content. Furthermore, qualifying institutions may support the Open Science movement and SJWEH by considering membership in NOROSH (4) (for further information see www.norosh.org/pmwiki.php/Main/Membership).
